# “It Takes A Decent Amount of Courage”: The State of Civics Education in Social Work Programs in Germany

**DOI:** 10.1007/s42972-022-00068-z

**Published:** 2022-11-21

**Authors:** Matthias Kachel

**Affiliations:** BayWISS Promotionskolleg Sozialer Wandel, Katholische Stiftungshochschule München, Munich, Germany

**Keywords:** Social work, Political social work, Policy practice, Civics education, Political education, Political participation, Civic participation, Schools of Social Work, Germany

## Abstract

There is an ongoing debate in German Social Work theory about whether Social Work is, can be, or should be a political profession. There are three opinions among scholars that answer either with a wholehearted “yes,” a skeptic “yes, but…,” and a resounding “no.” And even when the answer is yes, political activity of Social Workers is often described as “Einmischung,” which can be translated as “interference” or “meddling,” thus undermining the crucial role that Social Workers play in the welfare state. This debate affects not only academic discourse but also the education of Social Workers throughout Germany. There seem to be as many different approaches as there are schools of Social Work to teaching — or not teaching — civics and political skills to Social Work students — thus, political knowledge and interest, practical skills, preparation, and the ability to speak politically can differ dramatically among Social Workers — which, in turn, influences the ability and willingness for political action. This is also reflected in the low numbers of German Social Workers that are organized in unions. For my doctoral thesis, I have compared ten different Social Work curricula at as many Schools of Social Work in Germany. My goal was to find out whether and how future Social Workers are receiving — or are at least able to receive — training for political situations and political work, and whether the skills taught are those needed in practice. In the course of working on my dissertation, I let students of Social Work and Social Workers discuss my findings about “their” study program in focus groups and at the same time asked if the skills taught on paper are also the ones taught in reality — and whether they are the ones needed in practice. I wish to present my findings in this article.

## Introduction

Social Work researchers and theorists in Germany are involved in an ongoing debate about whether and how political Social Work can be and should be. There is considerable discussion about the amount of mandates a Social Worker has: Is it just one, the client, or is it two, the client as the recipient of their services and the state (or society) as the provider of those services, or is it even three mandates that the Social Work profession introduces itself as a guideline and principle to which Social Workers must adhere (Böhnisch, 1973; Staub-Bernasconi, 1998; Grunwald & Thiersch, 2004; Benz & Rieger, 2016)?

One of the problems of Social Work in Germany is that there is no one single trade union or entity like a bar association or a chamber of crafts that would be able to provide a mandatory code of ethics or similar binding guidelines for Social Workers to adhere to, but we will come to that later.

However, in current academic discourse, there is a clear tendency toward the politicization of Social Work that has been embraced by students, scholars, professionals, and profession-related political organizations in recent years. Political movements such as Fridays for Future and Black Lives Matter have provided numerous opportunities for young people to become politically engaged in their communities.

Kirk and Duschinsky in the UK as well as Kulke in Germany have shown in their studies that there is a high level of interest in politics among Social Work students, but that political activity is low. The phrase “I have come on this program to learn how to be politically minded” is not only the title of Kirk and Duschinsky’s research but it is also the description one of the students chooses to talk about his motivation to study Social Work. Both studies show that students often experience a sense of powerlessness and resignation after they experience the interconnection of Social Work and politics in their practice placements during their studies. While they show an eagerness to learn about being politically active, they often formulate (or show) a lack of ability to participate in political discourse (Duschinsky & Kirk, 2013) (Kulke, 2019).

One question that can be asked — and that I also ask in my doctoral dissertation — is what role does political education or civics education — that is, the teaching of knowledge about the political system and the acquisition of skills and methods for influencing politics — play in the curriculum of Social Work programs at German Schools of Social Work? Are the skills taught the skills that will be needed (and applicable) in practice?

In order to ask and answer this question, we must first take a look at what civics education looks like in Germany before students enter universities of applied sciences to study Social Work.^1^

According to the “3. Ranking Politische Bildung,” a study conducted by Mahir Gökbudag and Reinhold Hedtke in 2019 at the University of Bielefeld, civics education does not play a major role in the curriculum of secondary schools in Germany. Since the German education system is organized individually by each state rather than at the federal level, the curricula as well as the, courses and topics offered vary from state to state. Thus, each state also places a different emphasis on civics education and devotes different amounts of instructional time to politics, policy, and opportunities to participate in them. Although they are all critically low, they each offer a different — low — percentage: While the southern state of Bavaria allocated between 0.5 and 1.1% of secondary school time for civics, Nordrhein-Westfalen, a state in the middle-west of Germany, reserved between 3.8 and 4.4% of its curriculum to education about politics. While the first ranking took place in 2018 and this ranking is from 2019, new numbers suggest that there has not been much development to increase these numbers since then (Gökbudak & Hedtke, 2020).

So, if German schools provide so little civics education, where do young Germans get it from? One answer would be the parental home and family background — Bourdieu suggests that this is where most of the foundations for future education are laid, through the social capital provided by parents and extended families (Bourdieu, 1983). In addition, youth organizations and associations, “Jugendverbände”, provide informal education possibilities by offering activities where children, adolescents, and young adults can learn to apply and assert themselves in group activities. As mentioned earlier, movements such as Fridays for Future, Black Lives Matter, and other political youth organizations such as the youth groups of political parties — for example Junge Union, Jusos, Junge Liberale or Grüne Jugend — also offer similar group activities while influencing “adult” politics within the party (Massing, 2013).

While this sounds like a rich smorgasbord of informal education, it must be emphasized that while these offerings are in principle open to all, they are primarily used by young people with a certain access to education, monetary, or equivalent resources and privilege. Apart from that, gender is also a factor when it comes to actually becoming politically active — more boys and men are politically active and actively supported in their political development than girls and women (the German magazine “Katapult” opened their issue in September of 2020 with an article about the fact that there are more mayors named Thomas in Germany than there are female mayors.) (Keusch, 2020).

This suggests that civics education — or, as one of Duschinsky and Kirk’s participants puts it, “learning how to be politically-minded” — is an exclusive and underdeveloped educational process in Germany that, although there are many different actors and educators, does not reach all young citizens in Germany (Duschinsky & Kirk, 2013).

Furthermore, it seems reasonable to say that since there are different school systems in Germany, and there are many different possibilities to reach an education at a university of applied sciences, the educational “starting point” for students of Social Work differs extremely from student to student. This also applies to civics or political education, which lead me to the following questions while developing my research project:

- Is civics education content — that is, content aimed at teaching knowledge about the political system and skills for political action, as well as teaching both — embedded in the Social Work degree program?
- If so, how?
- Is the content that is embedded in module plans also the content that is taught in the degree program?
- Is the content that is taught during study also the content that is relevant to work in Social Work practice?

The purpose of this paper is to present the methodology used to answer these questions and the answers that resulted from its implementation. I will also show the conclusions and categories that emerged from this research project that makes up my PhD project.

## Methodology

My research design consisted of two steps: an analysis and comparison of ten Social Work module plans at ten different Schools of Social Work in Germany in combination with focus groups of students and alumni of the compared schools. The schools were selected based on their regional significance in agreement with my PhD supervisor. I chose one school for each region of Germany, east, west, south, and north. For that, I chose Leipzig University of Applied Sciences (HTWK) for the East, FH Münster University of Applied Sciences for the West, University of Applied Sciences (HAW) Landshut for the South, and Hochschule für Angewandte Wissenschaften (HAW) Hamburg for the North. To take into account and show possible differences within and between regions and different forms of organizational ownership, I have added six more courses: For example, to show differences between public and private education, I have considered the module plan of private University of Applied Sciences Fresenius. To compare the public Schools of Social Work with the numerous church-operated Universities of Applied Sciences that offer Social Work, I included the catholic KSH München University of Applied Sciences, Katholische Hochschule für Sozialwesen Berlin (KHSB), and Protestant University of Applied Sciences Bochum — which was also intended to serve as a comparison for a presumed gap between East and West Germany that may have remained from the German division into the Federal Republic of Germany and the socialist German Democratic Republic. Additionally, the difference between Universities of Applied Sciences under catholic and protestant management seemed interesting. To investigate if there are differences between urban and less urban environments, I added two further schools: Alice-Salomon-Hochschule Berlin, for a metropolitan but eastern setting that could also be comparable to an urban environment like Munich, and the smaller Merseburg University of Applied Sciences, which was supposed to provide a less urban, eastern setting.

### Curriculum Analysis

In the module plan analysis, returning to the research question of the importance of civics education in Social Work programs at the chosen Universities of Applied Sciences, I asked whether there are separate civics education modules or courses of civics education that belong to different modules, for example, that cover several in a single module. I also investigated how much time is available for studying these topics in class or for individual study.

### Focus Groups

In addition, I invited students and alumni to focus groups and let them discuss if the topics that these module plans provided were also the topics that were discussed in class and whether they felt adequately prepared for working politically in a professional Social Work setting. If they were already working in the field, the question became whether the political skills and knowledge awakened and fostered by the topics discussed in these educational settings were the ones that they could now use for their practice purposes and which ones they felt were needed. As a basic stimulus, they were confronted with the results of the aforementioned “3. Ranking Politische Bildung” to remind them of their own school time and to get them to talk about politics and their own experiences. They were then shown their School of Social Work’s module plan and the amount of time and topics devoted to civics education, with the additional questions, “What do you think about these topics? What would you have needed in addition or instead?” Two further follow-up questions addressed what they thought about the incorporation of civics education in their course of study in general and of the core curriculum provided by the Deutsche Gesellschaft für Soziale Arbeit (DGSA), (n.d.) the scientific organization of schools, teachers, and researchers of Social Work in Germany (DGSA). The last question gave the participants the opportunity to add topics not yet discussed and to give feedback to the interviewer.

This structure was adopted from Philip Mayring’s theory on qualitative content analysis. The chosen method of analysis was an interpretative-inductive form of interpretation, in which interpretative categories were developed from the collected research material (Mayring, 2002).

## Results

### Curriculum Analysis

As you can see in the graph below, of the ten chosen Universities of Applied Sciences, only four provided one or two modules explicitly dedicated to civics education for prospective Social Workers. Of the other six, five offered one or more (up to four) mandatory or voluntary courses that included civics education, while one offered none (while also not offering a dedicated module).

Apart from the fact that two out of the three catholic Universities of Applied Sciences offer a dedicated module for civics education but most of the state-sponsored ones did not, and that the Universities of Applied sciences in the North and South have dedicated modules while others do not, there appears to be no correlation between curriculum and religious or state sponsorship. Also, regional differences did not seem to play a role, at least not when looking at the curricula themselves.

Looking at the semester hours per week allotted to these courses, we can see that the amount of time spent on civics education in one of the ten examined programs ranges from 2 to 6 hours per week, which translates to approximately 30 to 96 hours of studies in the classroom plus additional hours of independent study for a semester length of 15 to 22 weeks. This means that in the ten Social Work programs selected for this analysis, an average of between 3.38 and 3.5 hours per week is spent specifically on civics education for a semester. The average Social Work degree program in Germany requires a course load that awards 210 credit points in the ECTS (European Credit Transfer and Accumulation System). Given the seven semesters most Social Work programs take, this means 30 credit points per semester. A 75-hour-module would be worth 7 or 8 credit hours, which is 26% of the total semester course load, or about a quarter of one seventh of the total Social Work course load. This means that civics education represents between 3.3 and 3.8% of the total coursework required to complete a bachelor’s degree in Social Work at one of the institutions analyzed in this PhD project.
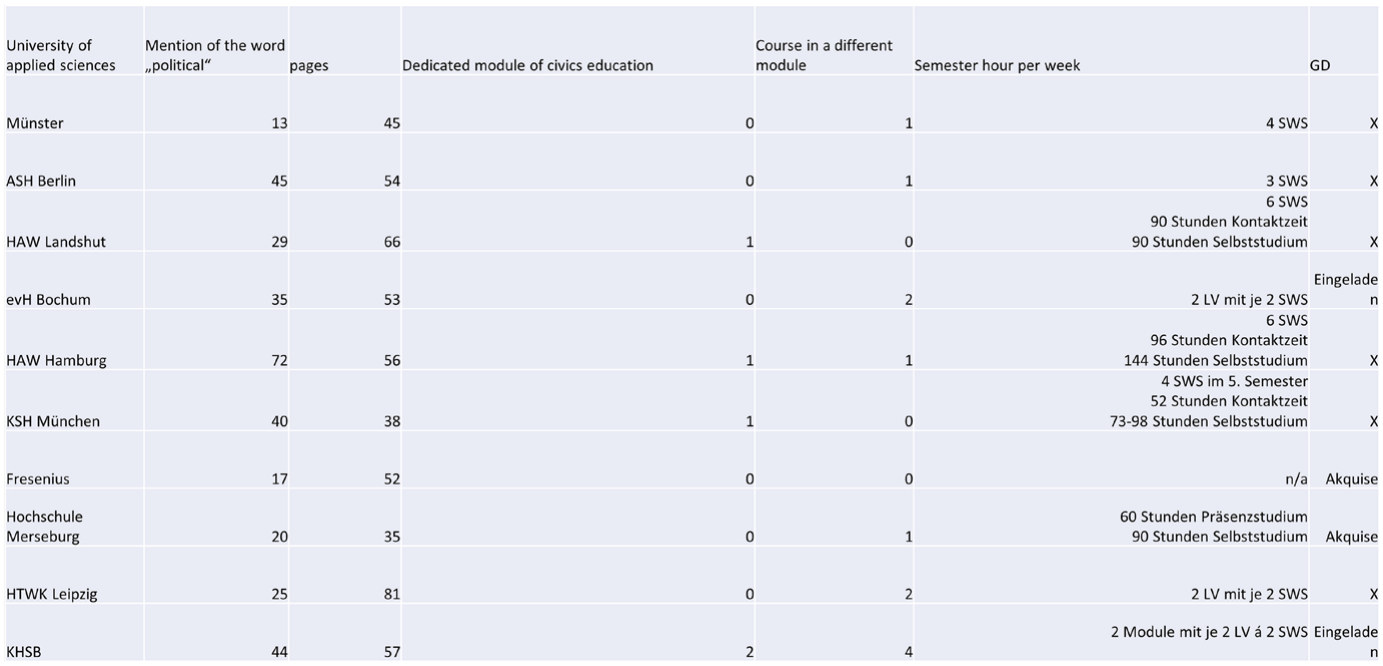


Interestingly, this number matches well with the figures provided by the “3. Ranking Politische Bildung” for secondary school education in Germany. It would be interesting to compare these numbers with those of Social Work programs in other parts of Europe or the USA.

### Focus Groups

Out of the ten examined programs, a total of six focus groups came together. For the interpretation of the existing focus groups, I chose to use the method of qualitative content analysis as described by Philipp Mayring (Mayring, 2002).

In this article, I would like to share some of the findings and categories that have emerged from the material:

#### “Speaking up (In Class) Takes Courage”

Several students stated in the focus groups that while the theoretical opportunity to engage in political discussions during and outside of class exists, that does not mean that every student feels comfortable engaging in the political arena. Much like the students in Kirk & Duschinsky’s findings, these students noted that on the one hand, they had learned to think and speak politically and felt a political interest, but on the other hand, they felt discouraged by the formal setting of lecture halls, the fact that public speaking was far outside their comfort zones, and by those students who were already “fluent” in the language of politics. They also emphasized that smaller, more intimate settings such as smaller student cohorts (for example master groups), work groups, and informal settings such as thematic salons or regulars’ tables (Stammtische) made it easier for them to open up and try out new thoughts and vocabulary to find their own political and professional voices.

#### Fluency in Political Language

One category that has already been alluded to at various points in this article (and directly above) is the ability to engage and take part in political discussions. Since all students come to University (of Applied Sciences) with different levels and forms of understanding of politics, they have different levels of ability to speak about it. Faculty and students likewise need to be aware and make sure that tertiary education is a place where political awareness and language skills can be acquired, refined, and practiced, with the clear need to be able to fail, to unintentionally say unwise or offensive things, in order to gather knowledge and feedback, and be able to adjust views and word choices accordingly. Education, even if it is at the university level, needs to be a form of laboratory or field experiment in which new skills can be tested.

#### Awareness of Individual Starting Points

Equally important, and already mentioned earlier, is the faculty’s awareness of the fact that due to the individual education backgrounds of Social Work students, everyone’s civics education history begins at a different point in their respective lives — while some have started long before entering University, others are just starting now and need either additional opportunities or a common starting point where everyone (re)begins in the context of the Social Work program. The students discussed their personal upbringing and backgrounds and where they first became interested in politics as well as when and how they achieved their own political language skills and unanimously agreed that this was an ongoing process to which different situations and contexts contributed, such as family, friends, education, and political or social activity.

#### Importance of a Balance Between Formal/Theoretical and Informal/Practical Learning Experiences

Many of the attending students and alumni spoke about their experiences with different kinds of teaching methods and activities while studying Social Work. Consistently, students said that they were more likely to recall learning experiences and lectures in which they had the opportunity to try out their new skills themselves or acquire them by practical means. For example, some students recalled role-playing experiences and model games in which they took on the roles of different political entities, decision makers, and interest groups to navigate and negotiate certain scenarios. Similarly, they emphasized the importance of experiences outside the formal lecture settings for the development of their political skills. They also suggested that informal (group) activities and study-related tasks such as organizing a panel discussion, developing programs, or fairs for certain days like World Social Work Day or World Human Rights Day are good learning environments that could be transformed into formal learning contexts and awarded with credit points or grades. In addition, the participants mentioned informal groups like trade union student groups, self-organized student groups for specific causes such as refugees as places and environments where they could test and further educate themselves.

#### “They Didn’t Teach Them That, They Just Did It Themselves” — The Importance of a University Campus’ Function As a Third Place and Faculty As Enablers of Action

One way to learn about politics that was mentioned repeatedly by students was through student government. On the one hand, they mentioned it as a way to influence the student body and faculty and bring about change; on the other hand, they spoke of the learning opportunity it provides by offering a wide range of political tools whose function can be learned by using them. Participants also discussed the spatial arrangements and accommodations a University of Applied Sciences provides as important for an informal learning experience. Is there undedicated space on campus that can be used by the students in their free time? Are there restrictions on how places can or should be used? Are there informal meeting places like dedicated rooms — for example, a student-organized bar, a student government office, spaces designated for informal and semi-formal group meetings to work on assignments or their own projects? Some students and alumni praised their University of Applied Sciences for providing space that students themselves could fill with meaning and purpose or turn into a sociological third place, a “home away from home” (Oldenburg, 1989). Others spoke of how the lack of such facilities also meant that informal learning experiences and political activities were absent. It seems that faculty and administration need to have an eye on providing possibilities for students to “appropriate” school resources for projects and personal development to create a true third place.

#### Topics That Do Not Occur in the Curriculum

When asked if the topics included in their module plan were the topics that they felt they would need or be able to use in their future work in the field, most participants confirmed that they felt these topics were necessary to provide them with a solid understanding and skills in political engagement. However, they also described that they felt certain topics were not included in the curriculum but should be because of their current or future relevance for the practice of Social Work. While some identified the digitalization of Social Work or responses to the current COVID-19 crisis as topics that they needed to know more about, all focus groups mentioned that their program did not provide them with enough skills and knowledge about postcolonial studies, the impacts of the climate crisis on Social Work, the tools to critically review Social Work concepts such as the triple mandate, or the idea of Social Work as a human rights profession.

#### Research Questions Revisited

At this point, it makes sense to take another look at the research questions posed at the outset in order to try to answer them concisely with the knowledge gained in the two steps of this research project:

*• Is civics education content – that is, content aimed at teaching knowledge about the political system and skills for political action, as well as teaching both—embedded in the Social Work degree program?*

**Yes, it takes up between 3.3 and 3.8% of the curriculum of the ten Social Work courses examined in this PhD project.**

*• If yes, how?*

**It takes place in form of individual courses in mixed modules and occasionally in separate modules — but for the most part in formal courses rather than through activities.**

*• Is the content of the module plans also the content of the course?*

**Yes, for the most part.**

*• Is the content that is taught in the course of study also the content that is relevant for work in Social Work practice?*

**To a large extent, but there is a need for updating content and more practical experience in political action.**

## Conclusions

The answers and results described above lead me to the following conclusions, which include recommendations and perspectives for Social Work education and its future development in Germany.

### Civic Education in Social Work Studies Can and Should Take Place in an Interplay of Formal and Informal Learning Contexts

A University of Applied Sciences cannot only be seen as a space for formal education and the imparting of theoretical knowledge. Civics education can also be taught through personal experiences and in a kind of laboratory or workshop setting where students have the opportunity to “try things out” — be it political speech or political action. These experiences can be shaped by the students themselves and accompanied by a member of faculty to provide advice and resources. In fact, these experiences may be preferable to teaching methods that convey theoretical information.

Given the right environment, a School of Social Work can also act as a Third Space that provides room, resources, and opportunities for informal political education and political action that can impact social development and change within and outside the institution. Consideration should be given to whether and how these informal courses of action can and should be implemented into formal learning processes or how they can be honored within the formal grading system of a School of Social Work. This also raises the need to think about how personal and professional development of students of Social Work can be assessed in the future, as a formalized and standardized grading system does not always reflect the development of the individual.

### Education at Universities of Applied Sciences Can Act Both as a “habitus reinforcer” and as a “habitus changer”

Schools of Social Work and their faculty should keep in mind that their students begin their tertiary education at very different levels of knowledge and skill sets regarding politics and civics. Given this, it seems necessary to establish a “status quo” from where students start their studies together, regardless of their current level, to allow them to learn together and from each other. Because students begin at these different points in their lives, they have different personal backgrounds and have had various individual opportunities to accumulate social capital (Bourdieu, 1983). Therefore, this should be considered when planning and implementing module plans.

Schools of Social Work as well as faculty, the student body, and external stakeholders such as trade unions, scientific organizations, or associations of professionals can support Social Work education with informal and formalized offers intended to either supplement and enhance existing module plans for civics education or to replace them. This also means that Schools of Social Work must be perceived as a public learning environment in which all organizations that employ and/or represent Social Workers should be able to provide curricular and extracurricular educational content. At the same time, Schools of Social Work must maintain as much independence as possible in order to remain a Third Space. This independence is necessary to create a neutral teaching environment that can be filled with the experiences and opportunities that informal learning contexts can provide. This does not necessarily mean that it should remain politically neutral — it means that the organization can be shaped, or at least influenced, by those currently studying Social Work to provide opportunities for additional generations of Social Work students to learn about politics and improve their own political skills.

### Final Thoughts

This means that, taking this line of thought to its extreme, Schools of Social Work and their faculty will need to be highly flexible organizations and individuals that provide their students with a unique and individualized study experience that requires a great deal of personal responsibility and independence and might be more comparable to membership in a democratically constituted association. This also means that the current form of higher education may need to undergo some significant changes.

While the last two thoughts bring the results of this PhD project to its furthest extreme outcome, we can conclude so far that the intersections between formal and informal education in Social Work as well as the role students play in their own educational process need to be looked at a bit more closely in Social Work programs in Germany in the near future.

I want to end by thanking Tobias Kindler and Rick Hoefer for the opportunity to participate in the 2021 International Policy Practice Meeting and for the invitation to write this article and contribute to the special issue of the Journal of Policy Practice and Research.

## Footnotes

1 Universities for the most part do not offer Social Work programs in Germany, instead — albeit sometimes — offering Social Pedagogy as an elective or as a specialization in the context of larger Pedagogy programs.
